# Three new “caecate” earthworm species from Sulawesi, Indonesia (Oligochaeta, Megascolecidae)

**DOI:** 10.3897/zookeys.805.24834

**Published:** 2018-12-11

**Authors:** Fahri Fahri, Rizki Amaliah, Bambang Suryobroto, Tri Atmowidi, Anh D. Nguyen

**Affiliations:** 1 Department of Biology, Faculty of Mathematics and Natural Sciences, Tadulako University, Jalan Raya Soekarno–Hatta, Tondo, Palu, 94117, Central Sulawesi, Indonesia Tadulako University Palu Indonesia; 2 Department of Biology, Faculty of Mathematics and Natural Sciences, Bogor Agricultural University, Dramaga Campus, Bogor 16680, Indonesia Bogor Agricultural University Bogor Indonesia; 3 Duy Tan University, 254, Nguyen Van Linh, Da Nang city, Vietnam Duy Tan University Da Nang Vietnam

**Keywords:** Caecate earthworms, Indonesia, *
Metaphire
*, new species, *
Pithemera
*, Sulawesi

## Abstract

Three new earthworm species are described from Sulawesi, Indonesia. Two belong to the genus *Pithemera* Sims & Easton, 1972, namely *P.suwastikai* Fahri, Amaliah & Atmowidi, **sp. n.** and *P.tadulako* Fahri, Amaliah & Atmowidi, **sp. n.** The new species, *P.suwastikai***sp. n.** is distinguished by a medium size (135–165 mm long, 4.5–6.5 mm diameter), four pairs of spermathecal pores in 5/6/7/8/9, 7–12 setae between male pores, no genital markings, holandry, and simple intestinal caeca. *Pithemeratadulako***sp. n.** is recognized by a large size (217–340 mm long, 13–15 mm diameter), two pairs of spermathecal pores in 7/8/9, no setae between male pores, no genital markings, holandry, and simple intestinal caeca. Another new species, *Metaphirerusydii* Fahri, Amaliah & Nguyen, **sp. n.**, is diagnosed by its large size (250–280 mm long,12–16 mm diameter), two pairs of spermathecal pores in 7/8/9, no setae between male porophores, presence of genital markings in the male region, holandry, and complex intestinal caeca. Additionally, an identification key to “caecate” species is provided to the Sulawesi’s fauna.

## Introduction

Earthworms with intestinal caeca consists of four genera belonging to family Megascolecidae, namely *Pithemera* Sims & Easton, 1972, *Amynthas* Kinberg, 1867, *Pheretima* Kinberg, 1867, and *Metaphire* Sims & Easton, 1972. This group is distributed in the Oriental to Australian region (Sims and Easton 1972). Of those genera, *Metaphire* can be easily recognized by the presence of intestinal caeca in segment xxvii, the presence of copulatory pouches, and no nephridia on spermathecal ducts (Sims and Easton 1972). *Pithemera* is recognized by paired male pores discharging directly onto surface of xviii, small spermathecal pores, 3–5 pairs between 4/5–8/9 and intestinal caeca originating in or near xxii, rarely xxiv, paired laterally or single mid-ventrally (Sims and Easton 1972). The genus is mainly distributed in the Solomon Islands, New Britain, Fiji, Samoa, and Philippines (Sims and Easton 1972). Although two genera *Pithemera* and *Metaphire* have been reported from various regions surrounding Sulawesi, for example, the Philippines, Malaysia, Japan, Vietnam, Taiwan, New Guinea and other parts of Indonesia (Ude 1905, 1932; Gates 1975; Benham 1897; Beddard 1899; Michaelsen 1899; Hong et al. 2008; Nguyen et al. 2016), they both have never been recorded in Sulawesi until this research.

Earthworms from Sulawesi have been reported by Perrier (1872), Fletcher (1886), Michaelsen (1891, 1899), Beddard and Fedarb (1895), and Benham (1896). All species belong to four genera, *Polypheretima* Michaelsen, 1934, *Planapheretima* Michaelsen, 1934, *Pheretima* Kinberg, 1867, and *Amynthas* Kinberg, 1867. Recently, Fahri et al. (2017) described more four new species of the genus *Polypheretima* from this region. However, regarding the caecate earthworms, there are no reports for this group from Sulawesi except Benham’s (1896). Our work herein contributes to a better understanding of earthworm diversity in Sulawesi though the description of three new species.

## Materials and methods

**Figure 1.**
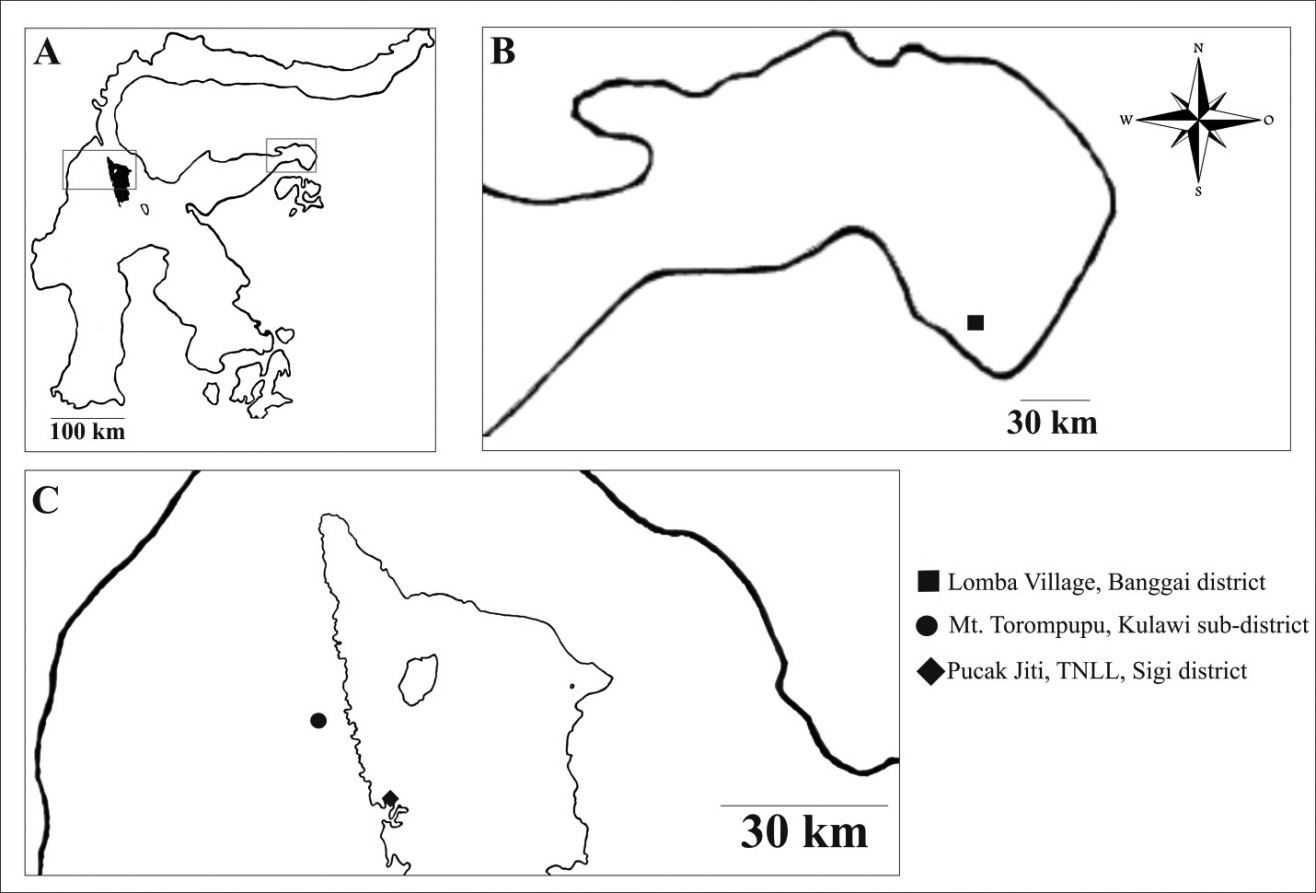
Location of earthworm study in Central Sulawesi Province. **A** Sulawesi Island **B** Banggai district, Central Sulawesi, **C**. Lore Lindu National Park, Central Sulawesi.

Fresh specimens were collected from yards at Lomba village (00°53'05.9"S, 123°17'50.0"E), Banggai district, 26 March 2016 and Puncak Jiti near Lore Lindu National Park (01°29'18.94"S, 120°02'09.28"E), Toro village, South Kulawi sub–district, Sigi district, Central Sulawesi, Indonesia, 28 May 2017. Hiking trail 1 (01°25'18.44"S, 119°55'00.14"E) 25 November 2017 and hiking trail No.2 (01°25'17.54"S, 119°53'53.4"E) 24 November 2017, natural forest of Mt. Torompupu, Quarlesi Mountain, Salua village, Kulawi sub–district, Sigi district, Central Sulawesi, Indonesia (Fig. 1).

The earthworms were killed in formalin 2%, transferred to 4% formalin for fixation for approximately 24 hours, and then transferred to fresh 4% formalin for long–term preservation and morphological studies. Specimens were dissected from the dorsal side for internal observation. Both external and internal morphology were observed under a Nikon type 104 C-LEDS stereo microscope. Holotypes and paratypes are deposited in Museum of Zoology in Bogor (**MZB**); some are housed in Tadulako University (**UNTAD)**.

## Taxonomic part

### Family Megascolecidae Rosa, 1891

#### Genus Pithemera

Taxon classificationAnimaliaHaplotaxidaMegascolecidae

Sims & Easton, 1972: 202

##### Type species.

*Perichaetabicincta* Perrier, 1875, by original designation.

##### Remarks.

*Pithemera* members are easily recognized by the origin of intestinal caeca at xxii. Sims & Easton (1972) divided *Pithemera* into three groups: *P.sedgwicki* group with intestinal caeca characters being single in mid-ventral, *P.bicincta* group with intestinal caeca being paired laterally and first spermathecal pores in 4/5, and *P.pacifica* group with intestinal caeca being paired laterally, first spermathecal pores in 5/6 and four thecal segments. Currently, the genus consists of 26 nominal species distributed mainly in New Guinea, and the Philippines (http://taxo.drilobase.org).

#### Pithemera suwastikai

Taxon classificationAnimaliaHaplotaxidaMegascolecidae

Fahri, Amaliah & Atmowidi sp. n.

http://zoobank.org/BBC7F172-0AA8-4468-9715-41ECF5A1B2A7

Fig. 2

##### Material examined.

**Holotype**. 1 mature (MZB Oli. 062), yards (00°53'05"S, 123°17'50"E), elevation of 5 m a.s.l., Lomba village, Banggai district, Central Sulawesi, Indonesia, 26 March 2016, coll. Mihwan Sataral, Sofyan Ladjatang, Endang Prasetyawati Wahyunigsih, and F. Fahri. **Paratypes**. 4 matures (MZB Oli. 063) and 3 matures (UNTAD Oli. 008), same data as for holotype.

##### Diagnosis.

Medium size, length 135–165 mm, diameter 4.5–5.5 mm at x and 4.5–6.5mm at xx, segments 110–127. Prostomium epilobous. First dorsal pore in 12/13. Setae 27–38 in v, 33–49 in vii, 38–54 in viii and 64–74 in xxv, and 7–12 between male porophores in xviii. Spermathecal pores arranged in four pairs, 5/6–8/9. Male pores paired, discharging directly onto surface of xviii. Genital markings absent. Holandric. Intestinal caeca originating from xxii, paired laterally.

##### Etymology.

The species is named after Dr I Nengah Suwastika, head of the Biology Department of Tadulako University for his kind support of our study on Sulawesi earthworms.

##### Description.

**Figure 2.**
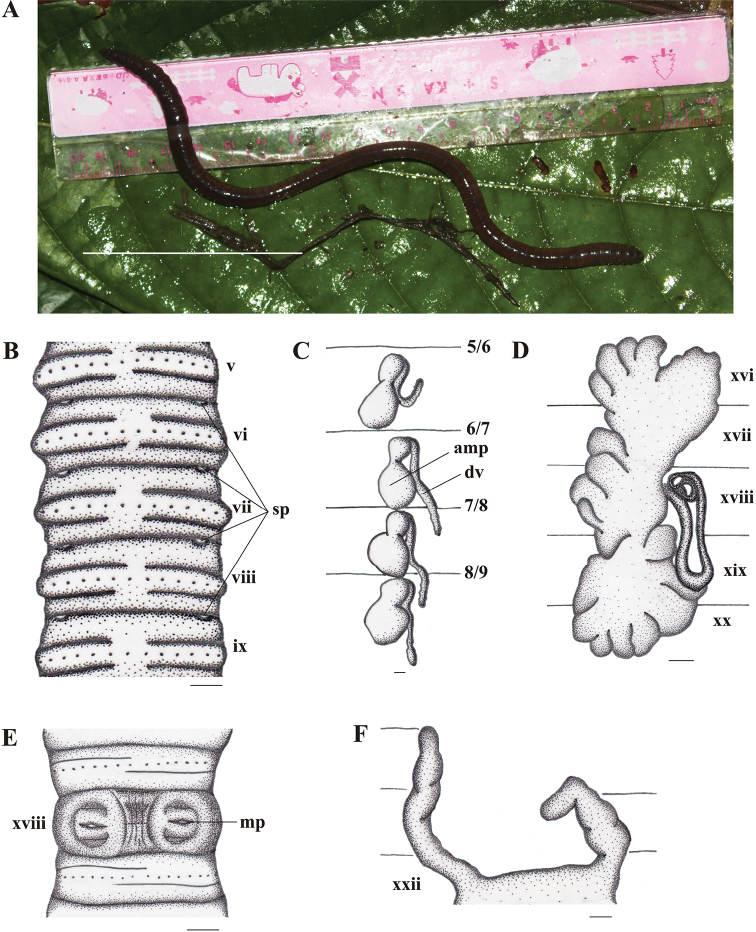
*Pithemerasuwastikai* sp. n., holotype. **A** Living specimen (dorsal view); **B** Spermathecal pore; **C** Spermathecae, right side on intrasegmental 5/6/7/8/9 (am = ampulla; dv = diverticulum); **D** Prostate gland; **E** Male pore region (mp = male pore); **F** Intestinal caecum. Scale bars: 50 mm (**A**), 1 mm (**B–F**).

*External characters.* Body generally cylindrical. Medium size, length 135–165 mm, diameter 4.5–5.5 mm at x and 4.5–6.5mm at xx, segments 110–127. Brownish red on dorsum, dark brown around clitellum and yellowish white on ventrum, especially in living specimens (Fig. 2A). After fixation, coloration brownish red on dorsum and whitish on ventrum.

Prostomium epilobous. First dorsal pore in 12/13. Setae regularly distributed around segmental equators: 27–38 in v, 33–49 in vii (Table 1), 38–54 in viii and 64–74 in xxv,7–12 between male porophores in xviii; setae distance aa=1–1.2ab, zz=1–3zy. Clitellum annular, xiv–xvi, smooth without setae and dorsal pores. Female pore single, mid-ventral in xiv.

**Table 1.** Character comparison among species of the *Pithemerapacifica*-group.

Character	*P.donvictorianoi* Aspe & James, 2015	*P.pacifica* (Beddard, 1899)	*P.dahli* (Ude, 1905)	*P.suwastikai* sp. n.
Length (mm)	41–58	56	70	135–165
Diameter (mm)	2.5–3.0	-	2.5	4.5–5.5
Segment number	85–100	90	120	110–127
Clitellum	xiv to xv	xiv–xvi/ xiv–xv*	xvi–xvi	xiv–xvi
Female pore	single	paired*	single	single
Spermathecae pore	5/6–8/9 (inconspicuous)	5/6–8/9	5/6–8/9	5/6–8/9
Dorsal pore	12/13	-	12/13	12/13
Copulatory pouches	absent	absent	absent	absent
Genital marking	absent	present in xii–xiii, xvii–xviii, xx–xxii	present in x, xi, xvii–xxi	absent
Setae on vii or viii	42–48 (vii)	47 (viii)	70 (viii)	33–49 (vii)
Setae bet. male pore	8	10	-	10–12
Septa	8/9 absent	8/9 present, thin	9/10 absent	8/9 absent
Intestinal caeca	xxii to xxi	-	-	simple (xxii–xx)
Last hearts	xiii	xii	xiii	xiii
Testis	holandric	metandric	metandric	holandric
Prostate gland	xvi to xix	xvii–xix	xvi–xxii	xvi–xx, xvii

*Note*: *: data from James et al. (2004).

Spermathecal pores small, slightly rounded, lateroventrally paired in 5/6–8/9 (Fig. 2A). No genital markings in the spermathecal region (Fig. 2B).

The openings of male pores small, on the setal ring of xviii, paired and discharging directly onto surface of xviii (Fig. 2E); ventral distance between male pores approx. 0.2x body circumference. Genital markings absent in the male region.

*Internal characters.* Septa 3/4/5/6/7/8 thick, 8/9 absent, 9/10/11/12/13 thin. Gizzard round within viii-x. Last hearts in xiii. Intestine origin at xv; caeca originating at xxii, extending anteriorly to xx, simple and paired laterally (Fig. 2F). Esophageal pouches absent. Typhlosole simple.

Four pairs of spermathecae paired in vi–ix. Ampulla round; duct enlarged, stout, approx. 1/4–1/2 as long as ampulla; no nephridia on spermathecal ducts. Diverticulum cylindrical, longer than ampulla, attached to base of ducts (Fig. 2C). No accessory glands.

Holandric. Testes sacs paired in x–xi, small, separated, ventral, yellowish. Seminal vesicles in xi–xii. Ovaries paired on xiii. Prostate gland racemose paired within xvi–xx or xvii–xx. Prostatic ducts long and U–shaped (Fig. 2D). No accessory glands.

##### Habitat and ecology.

Specimens were collected in yards at Lomba village, Banggai district, Central Sulawesi, Indonesia at 5 m a.s.l. The species was found in sandy soils at depths of 0–10 cm, around the roots of banana trees planted 1–2 m away from houses. No other specimens were collected despite continued collecting effort. The specimens were collected at an elevation of 50 meters above sea level. When collected, earthworms were moving actively on the ground.

##### Remarks.

This new species belongs to the *Pithemerapacifica* group characterized by having the first spermathecal pores in 5/6 and four thecal segments (Sims and Easton 1972). This group currently consists of three species, *P.pacifica* (Beddard, 1899) from New Guinea and Samoa, *P.dahli* (Ude, 1905) from New Guinea, and *P.donvictorianoi* (Aspe & James, 2015) from the Philippines. Although *Pithemerasuwastikai* sp. n. is classified into *Pithemerapacifica* group because of the presence of four thecal segments (5/6–8/9), the new species is totally differentiated from *P.pacifica* (Beddard, 1899) in having larger size (length 135–165 mm vs. up to 56 mm), holandric (vs. metandric), a single female pore (vs. a pair of female pores (see James et al. 2004)), clitellum in xiv–xvi without setae (vs. in xiv–xv/xvi with setae), absence of genital markings (vs. presence of genital markings).

The new species has similar characters to *P.donvictorianoi* Aspe & James, 2015, e.g., in the number of spermathecal pores, but different in clitellum (xiv–xvi vs. xiv–xv), setae on vii (33–49 vs. 42–48). *P.suwastikai* sp. n. also has different length with *P.donvictorianoi* (135–165 mm vs. 41–58 mm) and diameter (4.5–5.5 mm vs. 2.5–3.0 mm). In addition, *P.donvictorianoi* is a white worm, with small, narrow, oval spermathecal ampulla; ducts short, slender; diverticulum stalk short, attached to ducts, terminating in short ovate receptacle. In contrast, *P.suwastikai* sp. n. has brownish red pigment on dorsum, dark brown around clitellum and yellowish white on ventrum; ampulla rounded; ducts enlarged, approx. 1/4–1/2 as long as ampulla; diverticulum cylindrical, longer than ampulla, attached to base of ducts.

This new species is also similar to *P.dahli* (Ude, 1905) by having spermathecal pores in 5/6–8/9 and dorsal pore in 12/13. However, it differs from *P.dahli* in larger size (length: 135–165 mm vs. 70 mm; diameter 4.5–6.5 mm vs. 2.5 mm), absence of genital markings (vs. genital markings in x, xi, xvii–xxi), holandric (vs. metandric), and prostate glands in xvi–xx or xvii–xx (vs. in xvi–xxii). *P.suwastikai* sp. n. has also similar characters to *P.pacifica* (Beddard, 1899) in spermathecal pores (5/6–8/9) and dorsal pore (12/13), but it is totally different in absence of genital markings (vs. present in x, xi, xvii–xxi) and holandric (vs. metandric). The comparison of the four species is presented in Table 1.

#### Pithemera tadulako

Taxon classificationAnimaliaHaplotaxidaMegascolecidae

Fahri, Amaliah & Atmowidi sp. n.

http://zoobank.org/AA5F18DC-2EFC-4EC8-A5A9-3C9DDD08E4CC

Fig. 3

##### Material examined.

**Holotype**.1 mature (MZB Oli. 064), Puncak Jiti, natural forest, Lore Lindu National Park, Sigi district, Central Sulawesi, Indonesia (01°29'18"S, 120°02'09"E), elevation 1,370 m a.s.l., 28 May 2017, coll. Rizki Amaliah, Mus’af, Donny Aprilyanto. **Paratypes**. 2 matures (MZB Oli. 065), same data as for holotype. 1 mature (UNTAD Oli. 009), ca. 50 meters southwest of Kalimpa’a Lake (riverside entrance/inlet Kalimpa’a Lake), secondary forest, Lore Lindu National Park, Poso district, Central Sulawesi, Indonesia (01°19'33"S, 120°18'29"E), elevation 1,600 m a.s.l, 26 August 2017, coll. F Fahri, Sahlan, Evans Madiyono, Sucipto Suherman. 1 mature (**UNTAD Oli. 010**), ca. 50 meters north of the edge of Kalimpa’a Lake, around a water reservoir, same data as for paratypes, 11 November 2017, coll. Sahlan.

##### Diagnosis.

Large size, length 217–340 mm, diameter 13–15 mm, segments 120–123; darkish blue on dorsum, purplish brown around clitellum and yellowish white on ventrum. Prostomium epilobous. First dorsal pore in 12/13. Setae 42–56 in v, 48–65 in vii, 44–69 in viii and 55 in xxv, and no setae between male porophores in xviii. Spermathecal pores lateroventrally paired in 7/8/9. Male pores paired, discharging directly onto surface of xviii. Genital markings absent. Holandric.

##### Etymology.

The species is named after the well–known Tadulako University in central Sulawesi. ‘Tadulako’ means a ‘leader’ in indigenous culture of central Sulawesi.

##### Description.

**Figure 3.**
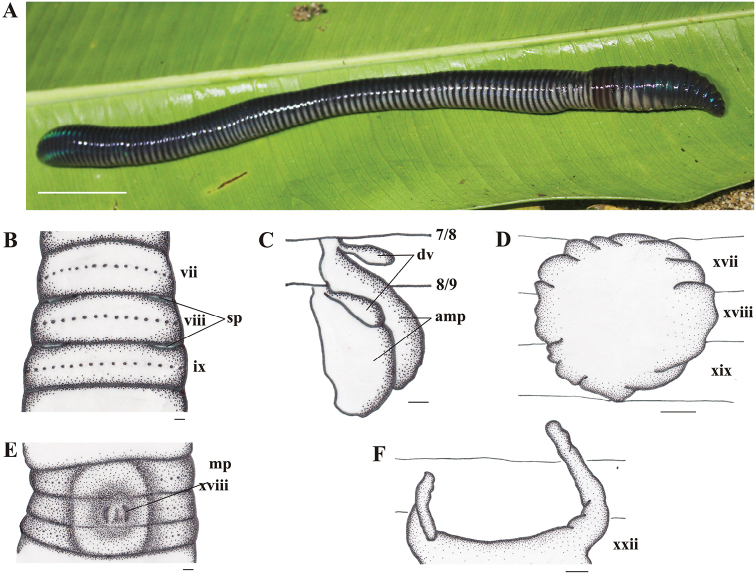
*Pithemeratadulako* sp. n., holotype. **A** Living specimen (lateral view); **B** Spermathecal pore; **C** Spermathecae, right side on intrasegmental 7/8/9 (am = ampulla; dv = diverticulum); **D** Prostate gland; **E** Male pore region (mp = male pore); **F** Intestinal caecum. Scale bars: 50 mm (**A**), 1 mm (**B–F**).

*External characters.* Body generally cylindrical; large size, length 217–340 mm, diameter 13–14 mm at x and 15 mm at xx, segments 120–123. Darkish blue on dorsum, purplish brown around clitellum and yellowish white on ventrum, especially for living specimens (Fig. 3A). After fixation, color grayish blue on dorsum and yellowish white on ventrum.

Prostomium epilobous. First dorsal pore in 12/13. Setae 42–56 in v, 48–65 in vii, 44–69 in viii and 55 at xxv, no setae between male porophores in xviii; setae distance aa=1–1.2ab, zz=1–2zy. Clitellum annular, xiv–xvi, smooth without setae and dorsal pores. Female pore single, mid-ventral in xiv.

Spermathecal pores small, lateroventrally paired in 7/8/9 (Fig. 3B). No genital markings in the spermathecal region.

Male pores small, forming vertical lines, paired and discharging directly onto surface of xviii (Fig. 3E); copulatory pouches absent; ventral distance between pores approx. 0.02x body circumference. Genital markings absent in the male region.

*Internal characters.* Septa 3/4/5/6/7/8 thick, 8/9 absent, 9/10/11/12/13 thin. Pharyngeal gizzard within viii–x. Last hearts in xiii. Intestine originating at xv; caeca originating at xxii, extending anteriorly to xx, simple (Fig. 3F). Oesophageal pouches absent. Typhlosole simple.

Two pairs of large spermathecae in viii–ix. Spermathecal ampulla oval; duct stout and very short; no nephridia on spermathecal ducts. Diverticulum claviform, much shorter than ampulla, attached to ducts (Fig. 3C). No accessory glands.

Holandric. Testes sacs paired in x–xi, large, separated, ventral, yellowish. Seminal vesicles in xi–xii. Ovaries paired on xiii. Prostate glands racemose, paired in xvii–xix (Fig. 3D). Prostatic ducts U–shaped, but invisible from dorsal view because of being hidden under prostate glands. No accessory glands.

##### Habitat and ecology.

Specimens were collected in Puncak Jiti, Toro village, South Kulawi sub-district, Sigi district at 1,370 m a.s.l. and near Kalimpa’a Lake, Lore Lindu National Park at 1,600 m a.s.l. This species was found on the surface of soils after rains that contains a lot of leaf litter.

##### Remarks.

The new species is totally different from species groups divided by Sims and Easton (1972) in first spermathecal pores in 7/8 and two thecal segments. The new species is clearly different from all other *Pithemera* species in its very large size (length 217–340 mm, diameter 13–14 mm at x and 15 mm at xx, segments 120–123), and color of living specimens (dark blue on dorsum, purplish brown around clitellum, and yellowish white on ventrum, color after fixation grayish blue on dorsum and yellowish white on ventrum).

The new species is somewhat similar to *P.viengthongensis* Hong &James, 2008 from Laos by having spermathecal pores in 7/8/9 and absence of genital markings. However, *P.tadulako* sp. n. is long (217–340 mm), and has more setae on vii (48–65), spermathecal ampulla being oval, ducts enlarged, short and stout; diverticulum cylindrical, shorter than ampulla. In contrast, *P.viengthongensis* is short (39 mm), has fewer setae on vii (37), spermathecal ampulla being ovate, smooth surface, duct short, thick; diverticulum stalk long and slender, chamber and chili-shaped. Moreover, two species are also distinguished by ventral distance between male porophores (0.02x body circumference in *P.tadulako* vs. 0.31x in *P.viengthongensis*).

#### Genus Metaphire

Taxon classificationAnimaliaHaplotaxidaMegascolecidae

Sims & Easton, 1972

##### Type species.

*Rhodopisjavanica* Kinberg, 1867, by monotypy.

##### Remarks.

This genus currently contains approximately 198 species widely distributed in the Oriental regions from Japan southwards through the Indo-Australasian archipelago to the rain forests of Australasia eastwards through Oceania (http://taxo.drilobase.org/index.php?title=Metaphire).

#### Metaphire rusydii

Taxon classificationAnimaliaHaplotaxidaMegascolecidae

Fahri, Amaliah & Nguyen sp. n.

http://zoobank.org/CB4326F8-A15E-46DA-8F37-D8DE58E828D0

Fig. 4

##### Material examined.

**Holotype**. 1 mature (MZB Oli. 066), hiking trail No.1 (01°25'18.44"S, 119°55'00.14"E), elevation of 1,120 m a.s.l., 25 November 2017, natural forest of Mt. Torompupu (Quarlesi Mountain), Salua village, Kulawi sub-district, Sigi district, Central Sulawesi, Indonesia, coll. Anna J Holmquest, F Fahri. **Paratypes**. 1 mature (UNTAD Oli. 011), hiking trail No.2 (01°25'17.54"S, 119°53'53.4"E), elevation 1,360 m a.s.l., 24 November 2017, same data as for holotype, coll. Jackie Childers & S Sarino.

##### Diagnosis.

Large size, length 250–280 mm, diameter 12–16 mm, segments 117–119; purplish blue on dorsum, and paler on ventrum. Prostomium 1/3 epilobous. First dorsal pore in 11/12. Setae 36–51 in v, 45–54 in vii, 41–59 in viii and 43–51 in xxv, and no setae between male porophores in xviii. Male pores located in xviii, within copulatory pouches. Genital markings present only in the male region. Spermathecal pores lateroventrally paired in 7/8/9. Holandric. Intestinal caeca complex or lobed mesially.

##### Etymology.

The species is named after Dr H M Rusydi Hasanuddin, Dean of Faculty of Sciences at Tadulako University, for his kind support to the authors for this research.

##### Description.

**Figure 4.**
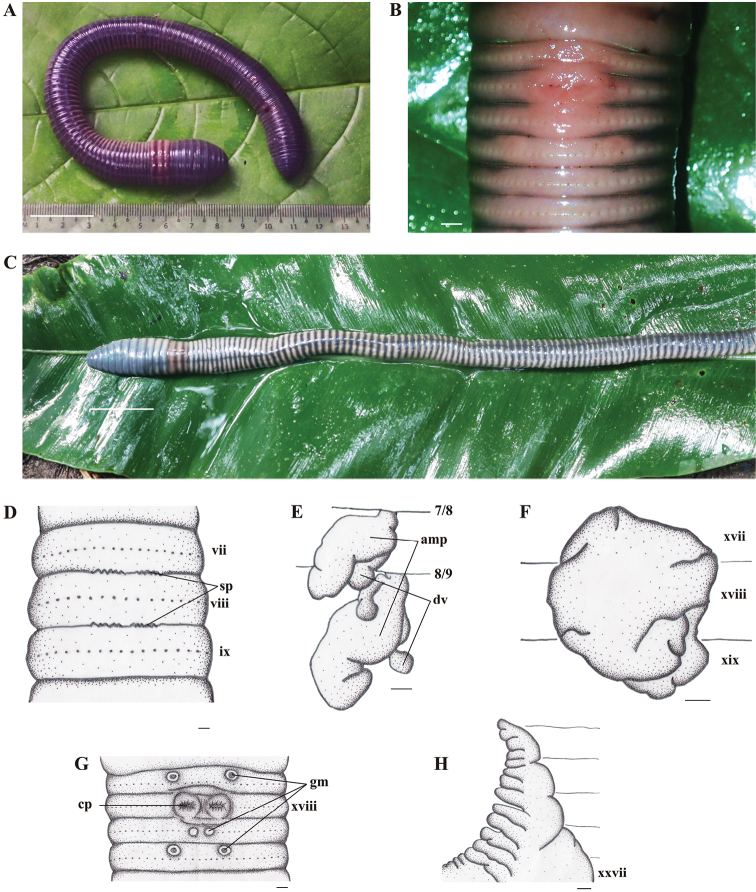
*Metaphirerusydii* sp. n., holotype. **A** Living specimen (dorsal view); **B** Living specimen (ventral view of male pore region); **C** Preserved specimen (dorsal view); **D** Spermathecal pore; **E** Spermathecae, right side on intrasegmental 7/8/9 (am = ampulla; dv = diverticulum); **F** Prostate gland; **G** Male pore region (cp = opening copulatory pouch); **H** Intestinal caecum. Scale bars: 50 mm (**A**), 1 mm (**C–H**).

*External characters.* Body generally cylindrical. Large size, length 250–280 mm, diameter 12–16 mm at x and 13–15 mm at xx, segments 117–119. Purplish blue on dorsum through the lateral region, clitellum purplish brown on dorsum through the lateral region and paler on ventrum, especially for living specimens (Fig. 4A–C). After fixation, color grayish blue on dorsum and yellowish white on ventrum.

Prostomium 1/3 epilobous. First dorsal pore in 11/12. Setae regularly distributed around segmental equators, 36–51 in v, 45–54 in vii, 41–59 in viii and 43–51 in xxv, no setae between male porophores in xviii; setae distance aa=ab, zz=1–1.2zy. Clitellum annular, within xiv–xvi, smooth without setae and dorsal pores. Female pore single, mid-ventral in xiv.

Spermathecal pores obviously visible, horizontally elongated, jagged, or small, ventrally paired in 7/8/9 (Fig. 4D). Ventral distance between spermathecal pores 0.04x body circumference, and horizontal length of pores ca. 2–3 mm.

Male pores deeply located inside copulatory pouches in the setal ring of xviii. The openings of copulatory pouches horizontally elongated, with swollen edges; ventral distance between male porophores 0.02x body circumference (Fig. 4G). Three pairs of small, rounded genital markings including two in front of the setal rings of xvii and xx, and one medially located in the setal ring of xix (Fig. 4G). Ventral distance between genital markings in xvii and xx about 0.07x body circumference, and less than 0.01x body circumference in xix.

Internal characters. Septa 3/4/5/6/7/8 and 10/11/12/13 thick, 8/9 membranous and 9/10 absent. Gizzard in viii. Last hearts in xiii. Intestine beginning in xv; caeca laterally paired, complex or lobed mesially, originating at xxvii, then extending anteriorly to xxii (Fig. 4H). Pharyngeal micronephridia developed on anterior faces of 5/6/7. Lymph glands not seen. Typhlosole simple, poorly developed.

Spermathecae paired in viii and ix. Spermathecal ampulla large, oval shaped; ducts stout and short, 1/5 as long as ampulla. Diverticulum cylindrical, much shorter than ampulla, attached to ducts; distal part enlarged to a rounded seminal chamber (Fig. 4E). No nephridia on spermathecal ducts. No accessory glands in the spermathecal region.

Holandric. Testes sacs paired in x–xi, large, separated, ventral, yellowish. The copulatory pouches hidden inside body wall. Seminal vesicles in xi–xii. Ovaries paired on xiii, oviduct and ovisac developing in xiii. Prostate glands racemose, paired in xvii–xix (Fig. 4F). Prostatic duct U–shaped, invisible from dorsal view since being hidden under prostate glands. No accessory glands.

##### Habitat and ecology.

Specimens were collected in Mt. Torompupu (Quarlessi Mountain), natural forest at 1,120 m a.s.l. and 1,360 m a.s.l. This species was found on soil surface in piles of leaf litter after rains. According to local hunters in Mt. Torompupu, the species can be mostly found after rains and usually observed on old trails in the forest. We did not find this species at an altitude of less than 1,000 m a.s.l.; presumably, the species may be distributed in an altitude of more than 1,000 m a.s.l.

##### Remarks.

The new species, *Metaphirerusydii* sp. n., belongs to the *schmardae*-group, which is characterized by two thecal segments, holandry, and having structure multiple/complex of intestinal caeca (Sims and Easton 1972). This new species is fairly similar to *M.schmardae* (Horst, 1883) by having spermathecal pores in 7/8/9, holandry, and complex intestinal caeca in xxvii. However, they are different in number of setae between male porophores (0 vs. 12–14 setae). *M.rusydii* sp. n. is also similar to *M.xuanlocensis* (Nguyen & Lam, 2017) by having first spermathecal pore in 7/8 and first dorsal pore in 11/12. However, *M.xuanlocensis* has only one pair of spermathecal pores (in 7/8) whereas *M.rusydii* sp. n. has two pairs of spermathecal pores in 7/8/9. This new species is somewhat similar to *Metaphireisselii* (Cognetti, 1908) by having small male pores and holandry. However, they are different in the position of male pores (xviii vs. xix), spermathecal pores (7/8/9 vs. 6/7–8/9) and first dorsal pore (11/12 vs. 12/13).

## Discussion

To date, a total of 17 earthworm species has been recorded from Sulawesi (Perrier 1872; Fletcher 1886; Beddard & Fedarb 1895; Benham 1896; Michaelsen 1891, 1899; Fahri et al. 2017). Almost all species are endemic to Sulawesi, except the cosmopolitan species *Polypheretimaelongata* (Perrier, 1872), *Polypheretimaeveretti* (Beddard & Fedarb, 1895), and *Pheretimadarnleiensis* (Fletcher, 1886). However, this number is still far from reflecting the earthworm diversity of Sulawesi due to lack of intensive surveys. More new species are certainly awaiting discovery.

### Key to caecate earthworm species recorded in Sulawesi, Indonesia

**Table:** 

1	Intestinal caecum, origin at xxii	**2**
–	Intestinal caecum, origin at xxvii	**3**
2	Spermathecal pores in 5/6–8/9	***Pithemerasuwastikai* sp.n.**
–	Spermathecal pores in 7/8/9	***Pithemeratadulako* sp.n.**
3	Copulatory pouches absent; one to six pairs of spermathecal pores	**4**
–	Copulatory pouches present; two or five pairs of spermathecal pores	**9**
4	Spermathecal pores from 7/8	**5**
–	Spermathecal pores absent or in3/4/5/6/7/8/9	**8**
5	One pair of spermathecal pores in 7/8	*** Amynthas zebrus ***
–	Two pairs of spermathecal pores in 7/8/9	**6**
6	Genital marking absent; the base with 12 secondary caeca	**7**
–	Genital markings present	*** Amynthas purpurus ***
7	Dark olive-green. Ampulla with globular sac, with narrow (undulating) diverticulum, expanded terminally	*** Amynthas jampeanus ***
–	Stone-grey to French-grey posteriorly. Ampulla with pyriform sac, long duct, receiving an undulating diverticulum, terminally dilated	*** Amynthas digitatus ***
8	Spermathecal pores absent	*** Amynthas bonthainensis ***
–	Six pairs of spermathecal pores in 3/4/5/6/7/8/9	*** Amynthas hexathecus ***
9	Spermathecal ducts with micronephridia. Three to five pairs of spermathecal pores in 4/5/6/7/8/9	*** Pheretima darnleiensis ***
–	Spermathecal ducts without micronephridia. Two two pairs of spermathecal pores in 7/8/9	***Metaphirerusydii* sp. n.**

## Supplementary Material

XML Treatment for Pithemera

XML Treatment for Pithemera suwastikai

XML Treatment for Pithemera tadulako

XML Treatment for Metaphire

XML Treatment for Metaphire rusydii
